# Type 1 innate lymphoid cells: a biomarker and therapeutic candidate in sarcoidosis

**DOI:** 10.1172/JCI183708

**Published:** 2024-09-03

**Authors:** Inchul Cho, Andrew L. Ji

**Affiliations:** 1 Department of Dermatology,; 2 Black Family Stem Cell Institute,; 3 Department of Oncological Sciences,; 4 Department of Cell, Developmental and Regenerative Biology, and; 5 Tisch Cancer Institute, Icahn School of Medicine at Mount Sinai, New York, New York, USA.

## Abstract

Sarcoidosis is an inflammatory disease characterized by immune cell–rich granulomas that form in multiple organs. In this issue of the *JCI*, Sati and colleagues used scRNA-seq and spatial transcriptomics of skin samples from patients with sarcoidosis and non-sarcoidosis granulomatous disease to identify upregulation of a stromal-immune CXCL12/CXCR4 axis and accumulation of type 1 innate lymphoid cells (ILC1s) in sarcoidosis. The accumulation of ILC1s in skin and blood was specific to patients with sarcoidosis and not observed in other granulomatous diseases. The authors used a mouse model of lung granuloma to show that ILCs contribute to granuloma formation and that blockade of CXCR4 reduced the formation of granulomas, providing a proof of concept that sarcoidosis may be treated by CXCR4 blockade to prevent the progression of disease in patients. These results suggest ILC1s could serve as a diagnostic biomarker in sarcoidosis and a potential therapeutic target.

## Introduction

Sarcoidosis is a multiorgan inflammatory disease characterized by the formation of granulomas, which are spatially organized clusters of immune cells, in peripheral organs including the lung, skin, and bone (1). The course of disease progression varies among patients, ranging from natural regression of the disease to the development of severe lung fibrosis, leading to fatality. Both genetic and environmental factors determine the course of disease (2, 3). However, the etiology of the disease is unknown, and there is a lack of specific treatment and diagnostic markers to identify sarcoidosis and discern patients at risk of developing advanced forms of the disease.

In severe cases, which involve manifestations in the lung, heart, and nerves, symptoms include cough, dyspnea, arrhythmias, seizure, fatigue, and loss of vision. These major organs are affected in approximately 30% of patients with sarcoidosis (4), which leads to a mortality of approximately 25% (5). Sarcoidosis-associated mortality almost exclusively occurs when these major organs are involved. There is, therefore, an urgent need to develop treatments and identify those at risk of developing cardiac, neuronal, or pulmonary sarcoidosis.

Diagnosing sarcoidosis remains challenging, as its clinical symptoms are highly similar to other autoimmune diseases (6). Nonspecific radiograph findings during diagnostic workup, such as lung fibrosis, have been mistaken for lung cancer, resulting in patients receiving unnecessary radiotherapy and chemotherapy (7). Thus, a positive diagnosis often requires long-term monitoring of patients who present with sarcoidosis-like symptoms, potentially delaying treatment that could alter disease course (8).

## Dissecting cellular cross-talk in sarcoid granuloma formation

Granulomas increase the local concentration and activity of immune cells and, therefore, the likelihood of a self-directed immune response. As such, existing treatments are targeted at inhibiting the formation of granulomas. Granulomas consist of multiple immune cells, including T cells, B cells, antigen-presenting cells, and stromal cells such as fibroblasts (9). The spatial architecture and cellular composition of granulomas are highly reminiscent of tertiary lymphoid organs that form in peripheral tissues in response to chronic antigen stimulation such as upon infection and during cancer progression.

**Figure 1:**
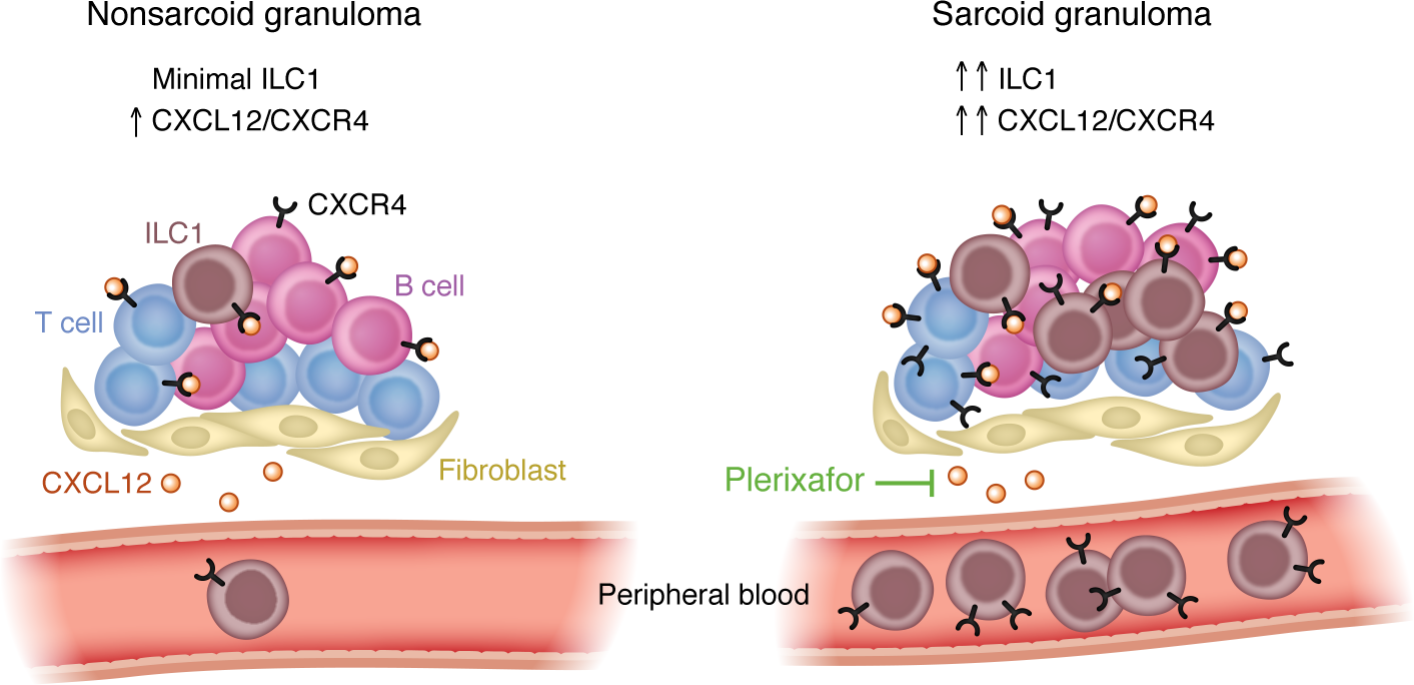
Diagnostic features of granulomas and peripheral blood indicate sarcoidosis and a target for intervention. Granulomas from sarcoidosis skin lesions possess higher expression of CXCR4 in T cells, B cells, and type 1 innate lymphoid cells (ILC1s) compared with granulomas from other skin diseases. Increased quantities of ILCs in peripheral blood of patients with sarcoidosis also indicate the potential of ILCs as a biomarker for sarcoidosis. Notably, plerixafor inhibits the CXCL12/CXCR4 axis and the formation of lung granulomas in a preclinical mouse model.

In this issue of the *JCI*, Sati and colleagues used single-cell RNA-seq (scRNA-seq) and spatial transcriptomics to determine that CXCR4 was highly and preferentially expressed in T cells, B cells, and type 1 innate lymphoid cells (ILCs) in sarcoidosis lesional skin relative to nonlesional skin (10). CXCL12, the cognate ligand for CXCR4, was highly expressed in fibroblasts and has previously been shown to be expressed in tertiary lymphoid structures found in the peripheral tissues of Sjögren’s syndrome and mucosa-associated lymphoid tissue in patients with lymphoma (11) and upon viral infection of mice (12). By employing spatial transcriptomics, Sati and authors found that CXCL12 expression was localized in the granulomas of sarcoidosis-affected tissues. Taken together, their results show that CXCL12-expressing fibroblasts in the granulomas may recruit CXCR4-expressing immune cells, resulting in the formation of granulomas. Administration of an approved CXCR4 inhibitor, plerixafor, inhibited the formation of pulmonary granulomas in mouse models, suggesting that such therapeutic intervention may be possible in patients with sarcoidosis (Figure 1) (10).

The mechanisms of protection against granuloma formation by CXCR4 inhibition remains to be further explored. The authors argued that CXCR4 inhibition leads to reduced ILC1 accumulation, and in turn, reduced granuloma formation. However, given that CXCR4 can induce chemotaxis in T cells and B cells (both express higher levels of CXCR4) (13, 14), it is plausible that these immune cell subsets have a greater contribution to sarcoidosis. Indeed, the authors show a drastic decrease in granuloma formation in T cell– and B cell–deficient *Rag2*-knockout (*Rag2*-KO) mice compared with wild-type mice, and a more modest decrease between ILC-deficient (*Il2rg*-KO) and *Rag2*-KO mice. As such, it may be argued that granuloma formation, and CXCR4 inhibition, is largely driven by T cells and B cells, and less so by ILCs. The authors also identified lymphotoxin A^+^ and lymphotoxin B^+^ T cell subsets, which may home to CXCL12-rich regions and exacerbate the expansion of granulomas by inducing further expression of chemokines (15, 16). Addressing these questions are technically challenging due to a lack of models that can selectively deplete ILCs.

## ILCs as biomarker for sarcoidosis

The ILCs are akin to T cells. They derive from common lymphoid progenitors during development, which can also give rise to T cells and B cells (17). Similarly to T cells, ILCs can be functionally divided into type 1, 2, and 17 according to the expression of transcription factors (T-bet, Gata3, and Rorγt) and characteristic cytokines (IFN-γ, IL-13, and IL-17). In contrast to T cells, these cells lack the expression of lineage-defining markers (such as CD3, CD4, and CD8 for T cells).

Sati and authors used scRNA-seq and a dedicated panel of antibodies for the identification of ILCs. Blood from patients with sarcoidosis contained higher quantities of ILC1s than that from healthy volunteers or from patients with other granulomatous disease. The addition of ILC1 as a biomarker for sarcoidosis provides another parameter to distinguish sarcoidosis from other granulomatous diseases (10). We anticipate that the incorporation of lineage-negative markers and CD45 staining to routine histological analysis of biopsy samples and profiling of blood samples will be a welcome addition to the arsenal of diagnostic markers. However, addition of circulating ILC1 as a biomarker may be hindered by the requirement for a well-trained flow cytometrist.

## Single-cell and spatial transcriptomic profiling for discovery

Sati and colleagues leveraged single-cell and spatial transcriptomics to dissect the cellular composition of granulomas and the blood from patients. Through these approaches, the authors identified CXCL12/CXCR4 signaling as the driver of ILC recruitment. These approaches enabled quantification of cell type abundance, profiling of transcriptomes, and prediction of intercellular communication, particularly of a rare cell type such as ILC1s (10). Spatial transcriptomics approaches complement existing scRNA-seq approaches by revealing locations of cell types and their transcripts in situ. The addition of spatial information allows for cell-cell interactions to be ranked according to spatial proximity of cell types. Similar approaches have shown to be highly useful for understanding the etiology of other human diseases (18–21).

Continuing development in single-cell technologies and framework of analysis is expected to uncover hitherto unknown biology of numerous diseases. Early spatial transcriptomics tools, including Visium technology used by Sati and colleagues, are limited by poor resolution of 55 μm spot sizes. At such relatively low resolution, single cells (with a diameter of approximately 10 μm) cannot be resolved. Improvement in resolution with imaging technologies such as MERFISH, CosMx, or Xenium, or sequencing-based Stereo-seq or Visium HD offer subcellular resolution, allowing transcripts to be attributed to individual cells (22, 23). In parallel, as new frameworks for spatial data analysis develop (24), we are beginning to better understand spatially dependent cellular interactions that dictate responsiveness to cancer immunotherapy (25), tumor microenvironment (26), and colitis (27). The incorporation of single-cell-resolution spatial transcriptomics is likely to provide an unprecedented level of detail of pathological immune cell interactions in situ.

## Conclusion

Sarcoidosis is a life-threatening disease that can be managed if detected early. However, the road to diagnosis is complicated. Patients often present with symptoms that also manifest in other immunological diseases. Sati and authors have used unbiased transcriptomic profiling to identify ILC1s as a promising marker that can potentially aid diagnosis. Furthermore, they showed that an FDA-approved CXCR4 inhibitor, plerixafor, can inhibit the formation of granulomas in clinically relevant mouse models (10). Such bedside-to-bench approaches are poised to yield similar promising strategies for diagnosing and treating other intractable diseases. We hope that clinically relevant and translational insights will continue to accumulate with improvements in single-cell and spatial transcriptomic technologies and their ease of use.

## Version 1 09/03/2024

Electronic publication
